# Experiences and Implications on Family Relationships for Grandmothers Raising Their Grandchildren

**DOI:** 10.1093/geroni/igaa057.1108

**Published:** 2020-12-16

**Authors:** Carol Musil, Elizabeth Tracy, Rashon Braxton, McKenzie Wallace, Alexandra Jeanblanc

**Affiliations:** Case Western Reserve University, Cleveland, Ohio, United States

## Abstract

In the U.S., over 2.7 million grandparents are primary caregivers to grandchildren. It is critical to understand the experiences of grandparent caregivers to design tailored, supportive programs. Our aim was to analyze written journals of 129 grandmothers with respect to the impact of raising grandchildren on relationships with family members. As part of a national RCT study of grandmother caregivers, participants completed daily journals for 4 weeks. Employing thematic data analysis, three members of the research team coded using NVIVO 12 Plus. The research team met regularly to compare and resolve discrepancies in coding. Percent agreement was > 80%. Relationships with the grandchild’s mother were characterized by anger/tension, resentment, and the realization that the mother was incapable of parenting while at the same time expressing worry/concern for her. Relationships with the grandchild’s father mirrored these dynamics while also depicting the father as a distant figure, inconsistent, and financially absent. Spousal relationships were marked by challenges faced by the spouse, their shared role/influence as a grandparent, and the quality of their time spent together. The other relationships described were often focused on their other adult children, as well as their own adult siblings. Grandmother caregivers expressed stress or strain and frustration within these relationships. Caregivers also verbalized gratitude for support from family members within the context of familial and financial stress. Grandmother caregivers may need support in managing familial relationships and stress within these relationships, which may be a target for future interventions.

